# Supplementary material for: SurVIndel2: improving copy number variant calling from next-generation sequencing using hidden split reads
Source: Nat Commun. 2024 Dec 2;15:10473. doi: 10.1038/s41467-024-53087-7 (PMC11612505; doi:10.1038/s41467-024-53087-7)
Supplement: Supplementary file 1 — Supplementary Information [file 41467_2024_53087_MOESM1_ESM.pdf]

Supplementary Information

SurVIndel2: improving copy number variant calling from next-generation sequencing using hidden split reads

Supplementary Methods

Clustering split reads

We aim at clustering clipped reads and hidden split reads and, for each cluster of size at least three, generating a consensus sequence. Each consensus sequence represents a potential breakpoint of a CNV, and it is marked as right-clipped or left-clipped.

In the current algorithm, clipped reads and hidden split reads are not clustered together; in other words, a cluster cannot contain both clipped reads and hidden split reads. Left-clipped (resp. right-clipped) reads that are clipped at the same position (with a tolerance of 3 bp) are clustered together.

For hidden split reads, we heuristically determine whether a read is left or right-clipped as follows. We divide it into two halves, and count the number of differences with the reference (defined as number of mismatches plus the number of indels) for the left and the right half. If the left half has a higher number of differences than the right half, we mark the read as left-clipped; otherwise, we mark it as right-clipped. Left-clipped (resp. right-clipped) hidden split reads are clustered so that the overlap within any pair of reads within a cluster is at least half the read length.

The consensus generation proceeds identically whether the reads in the cluster are clipped or hidden split reads. For each cluster of size at least three, a consensus sequence is generated by casting a majority vote base by base [1]. Then, every read in the cluster is realigned against the consensus sequence: if the alignment contains any indel or the fraction of mismatches is greater than ϵ (ϵ is a user-defined value that represents the maximum expected fraction of sequencing errors in a read, 0.04 by default), the read is removed from the cluster. If less than three reads are retained, the whole cluster is discarded. Otherwise, a new, final consensus sequence is generated from the retained reads. Left-clipped (right-clipped) reads will produce a left-clipped (right-clipped) consensus.

Finding a putative range for breakpoints

As mentioned in the main text, the consensus sequence of a cluster of split reads represents a potential breakpoint of a CNV. Since a CNV has two breakpoints on the reference, we are interested in defining a range where to search for the second breakpoint. Fig. 1 shows how to calculate the range from a single right-clipped read. In order to calculate the range for a right-clipped cluster, we

simply calculate the intersection of the ranges of the individual reads. The calculation of the range for left-clipped clusters is simply symmetrical.

Generating the junction sequence

Given a right-clipped consensus sequence $R[1..r]$ and a left-clipped consensus sequence $L[1..l]$, we generate a junction sequence as follows. Let n be the largest integer such that the hamming distance between $R[r-n+1:r]$ and $L[1:n]$ is at most $n \cdot \epsilon$. If $n \geq 15$, the junction sequence is formed by concatenating R and $L[n+1:l]$; otherwise, no sequence is generated.

Extending a consensus sequence

Consensus sequences obtained by split and hidden split reads tend to be short, often not much longer than the length of a read. For this reason, when realigning them to repetitive regions we may obtain incorrect or ambiguous locations. By extending the consensus sequences using assembly we aim at improving the confidence of the realignment. This happens in two steps: first, a set of target reads are identified; second, the target reads are used to extend using an overlap-layout-consensus approach.

In order to detect the target reads, we first identify a target region where the desired reads are likely to be mapped. The target region is calculated as follows:

- If we are extending a right-clipped consensus to the left, the target sequence is the *maxIS* bp region left-flanking the consensus;
- If we are extending a left-clipped consensus to the right, the target sequence is the *maxIS* bp region right-flanking the consensus;
- If we are extending a right-clipped consensus to the right or a left-clipped consensus to the left, the target region is the opposite breakpoint range.

Then, all reads mapped within the target region are target reads. Furthermore, we search for pairs such that a read R_1 is aligned to the forward (resp. reverse) strand within *maxIS* bp upstream (resp. downstream) of the target region and their mate R_2 is aligned to a different genomic location: R_2 is added to the target reads (Supplementary Fig. 2).

Next, we use the target reads to extend the consensus. A directed graph is built where each node is a read, and an edge connects two reads $R_1 \rightarrow R_2$ if a suffix of R_1 and a prefix of R_2 of at least read length/2 bp are identical. The consensus sequence is treated as a read. If the connected component containing the consensus sequence contains a cycle, we remove every edge that touches a node in the cycle. Then, we find the longest path either starting from or ending at the consensus sequence, depending on whether we are extending the consensus to the left or to the right.

Clustering discordant pairs

Given a set of discordant read pairs, we want to partition them into clusters so that all read pairs in a cluster support the same CNV. For this purpose, we

use the algorithm used by SurVIndel [1]. Here we provide a brief, high-level description.

Each cluster is composed of two segments, a forward and a reverse segment. We define a merging operation between two segments as follows: given two segments $S_1 = (s_1, e_1)$ and $S_2 = (s_2, e_2)$, we create a new segment $S_m = (min(s_1, s_2), max(e_1, e_2))$. We then define a merging operation between two clusters by merging the two forward segments and the two reverse segments. We also define the distance between the two segments S_1 and S_2 is defined as $max(e_1, e_2) - min(s_1, s_2)$, and the distance between two clusters as the maximum distance between (a) the distance between the two forward segments and (b) the distance between the two reverse segments.

The algorithm proceeds as follows. Initially, each individual read pair forms a cluster, and its forward (resp. reverse) strand read is the forward (resp. reverse) segment of the cluster. Then, we keep merging the pair of clusters with the shortest distance, as long as the distance is less than $maxIS$. Intuitively, if a segment is longer than $maxIS$, it means that its cluster includes two reads that are more than $maxIS$ bp apart. Therefore, it is unlikely that such reads support the breakpoint of the same CNV.

Insertion size-based features

As mentioned in the main text, CNVs will distort the insert size of read pairs. In particular, a read pair with a fragment size L crossing a deletion of size d will be mapped to the reference with an insert size of $L + d$; when d is large, the read pair will be clearly discordant. However, when d is small, determining that the pair supports the presence of a deletion is not as easy. Note that we define a read pair as discordant when the insert size is greater than $max_{IS} = \mu + 3\sigma$. Consider a read pair that contains a deletion, and assume its fragment size is μ bp. If the deletion is less than 3σ bp, the read pair will not be detected as discordant.

More precisely, define $min_{IS} = \mu - 3\sigma$ to be the minimum acceptable insert size for a read pair. Remember that max_{IS} was defined similarly as the maximum acceptable insert size for a read pair. Then, when the deletion is of size greater than $max_{IS} - min_{IS} = 6\sigma$, we expect that all pairs containing the deletion can be identified as discordant, and we use a positive-to-negative ratio. Let P and N be the set of discordant and concordant (i.e., not discordant) pairs that contain the midpoint of the tested deletion. We compute the positive-to-negative ratio as $|P|/(|P|+|N|)$, and we reject the deletion if it is too low (lower than 0.25 by default).

However, this approach cannot be used for smaller deletions. This problem was tackled in SurVIndel [1] by analysing the distribution of all pairs that potentially contain the deletion. Although it may be impossible to determine whether a single pair supports the deletion or not, we expect the read pairs to have a larger insert size, on average, than a set of pairs that do not contain a deletion. Therefore, statistical tests are employed to determine whether a set of reads may contain a deletion.

Here, we calculate two features using two different tests. Both tests have two ingredients: a distribution C of read pairs that do not contain a deletion, and a distribution D of pairs that contain the candidate deletion that is being tested. C is generated by choosing 1 million random locations in the genome

and sampling the pairs that contain any of those locations. D is the set of read pairs that contain the midpoint of the deletion. The first test determines a confidence interval for the size of the deletion, by computing a 99% confidence interval for the difference of means between D and C, and tests whether the size of the predicted deletion falls within this range. The second tests employs a Kolmogorov-Smirnoff test to calculate a p-value estimating whether C and D are significantly different. The lower the p-value, the more likely C and D are different.

For the first test, the feature we obtain is 0 if the size of the deletion falls within the confidence interval, otherwise it is a positive number indicating how far outside the confidence interval the deletion falls. The second feature is the p-value of the KS-test.

The statistical tests are not currently applied to tandem duplications, because the distribution of reads pairs is far more complicated and challenging to use.

Filtering module training

The filtering module uses a random forest to filter putative false positives. The random forest can be trained on a given set of samples. For each sample, the module requires three files: the raw call (i.e., unfiltered) by SurVIndel2, the BAM statistics (generated by SurVIndel2) and a list of SV IDs that are believed to be false positives. A set of these files for the HGSVC2 samples are distributed with the software, and can be easily used with the provided scripts in order to build the model.

For the experiments in the paper, we used a leave-one out strategy. More precisely, for each sample S in HGSVC2, we trained a separate model by using all of the samples in HGSVC2 except for S. Then, we used this model solely to filter the raw calls of S. We employed the same approach when calling CNVs for the 7 Arabidopsis Thaliana samples.

Supplementary Figures

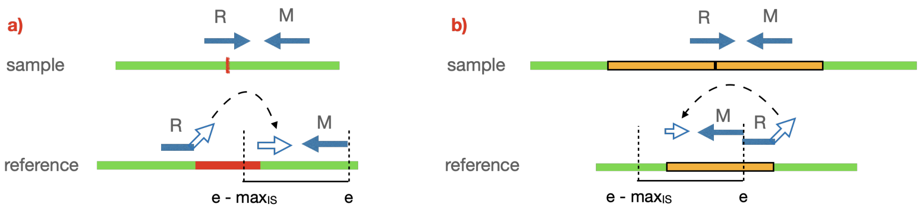

Supplementary Figure 1: **Determining a range for remapping the clipped sequence of a consensus.** (a) An example of a right-clipped reads R and its mate M. R determines the left breakpoint of the deletion, and remapping the clipped sequence can determine the right breakpoint. By the definition of read pair, the right half of R and M must be within max_{IS} bp of each other; therefore, we define as the acceptable range for the right breakpoint of the deletion to be $[e - max_{IS}..e]$, where e is the endpoint of M. (b) For duplications the situation is similar, except that the R determines the right breakpoint, and remapping the clipped sequence determines the left breakpoint. The acceptable range for the left breakpoint is therefore $[e - max_{IS}..e]$, where e is the endpoint of M.

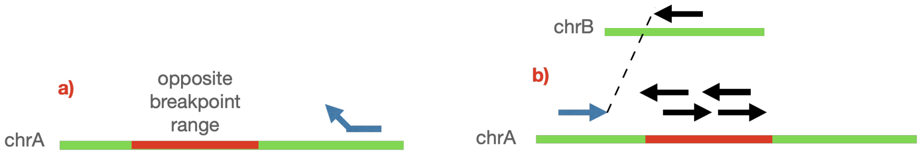

Supplementary Figure 2: **Detection of the target reads for the extension of a consensus sequence.** (a) First, we identify the target region that contains the reads we will use to extend the consensus. In this case, since we are extending a left-clipped consensus to the left, the target region (in red) is the opposite breakpoint range of the consensus. (b) Next, we collect the reads within the target region (reads in black). Furthermore, an additional read is collected from an entirely different genomic location (described as chrB in the figure). This is because the location of its mate (in blue) suggests that the read might belong to the target region.

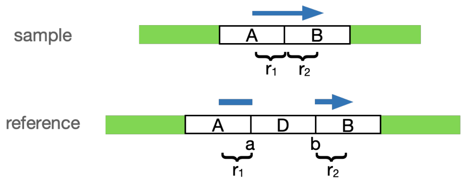

Supplementary Figure 3: **How deletions may generate split reads.** A region D, covering the reference from position a to position b, is deleted. A and B are the left- and right-flanking regions of D, respectively. In the sample genome, A and B are adjacent. Therefore, a read R of r-bp may be sequenced so that its r_1-bp long prefix is sequenced from A and its r_2-bp long suffix is sequenced from B (clearly, $r_1 + r_2 = r$). When R is aligned to the reference, it will not completely align to any location. Rather, its first r_1-bp will align to the r_1-bp long suffix of A, and its last r_2-bp will align to the r_2-bp long prefix of B.

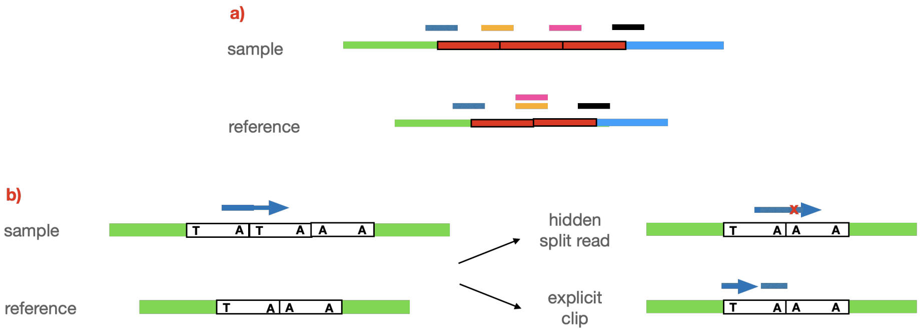

Supplementary Figure 4: **Duplications often do not generate split reads, but they may generate hidden split reads.** (a) Assume there is a tandem repeat with $n > 1$ repeat units in the reference, its repeat units are all identical, and its length is greater than the read length. If the same tandem repeat in the sample has $m > n$ repeat units, no split read will support the duplication. (b) Similarly to deletions, when copies of a tandem repeat are not identical, a duplication may generate hidden split reads.

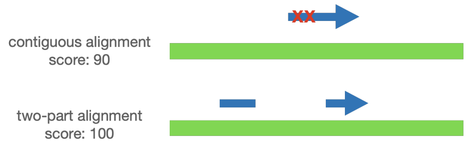

Supplementary Figure 5: **Example of HSR-score calculation.** A read is aligned to the reference with two mismatches. Suppose we assign +1 to a match and -4 to a mismatch; its alignment score will be 90 (98 matches and 2 mismatches = 98 - 8 = 90). If we allow the read to split into two, and its two parts to align independently without penalty, we can align both parts without mismatches. Therefore, the score is 100 (100 matches). The HSR-score of this read is 100 - 90 = 10.

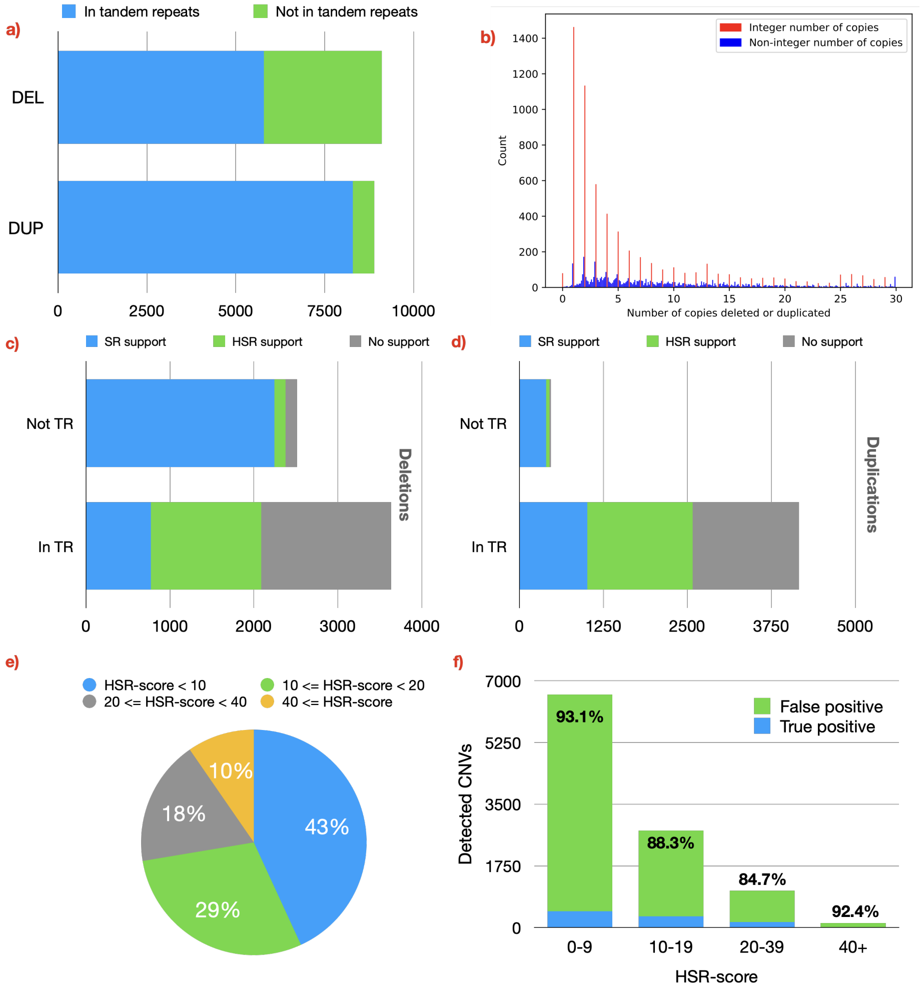

Supplementary Figure 6: **The impact of deletions deleting full repeat units and the importance of hidden split reads in detecting such deletions, studied on HG00512.** (a) We consider a deletion to be contained in a tandem repeat if at least 90% of the deletion is covered by a repetitive region. Nearly two thirds of the deletions in this dataset are contained in a tandem repeat. (b) For every deletion that overlaps with a tandem repeat, we calculated the number of repeat units that it deletes by dividing the length of the deletion by the length of the repeat unit. Bars representing an integer number of copies (i.e., the length of the deletion is multiple of the length of the repeat unit) are coloured red. Most deletions delete an integer number of repeat units. (c,d) Percentages of deletions outside (c) and contained (d) in tandem repeats that are supported by split reads, by hidden split reads, and by neither. Hidden split reads have the potential to recover a significant number of deletions missed by regular split reads. (e) HSR-scores of the hidden split reads supporting the existing copy number variants (CNV) in HG00512. Most reads have low (<20) HSR-score. (f) We found all hidden split reads in 10,000 randomly sampled repetitive regions, and for each we tried to predict a CNV by finding the optimal split alignment. Most CNVs (>91%) detected this way were false positive. This shows that using hidden split reads to detect CNVs is challenging, and effective filters must be employed to distinguish real from false CNVs.

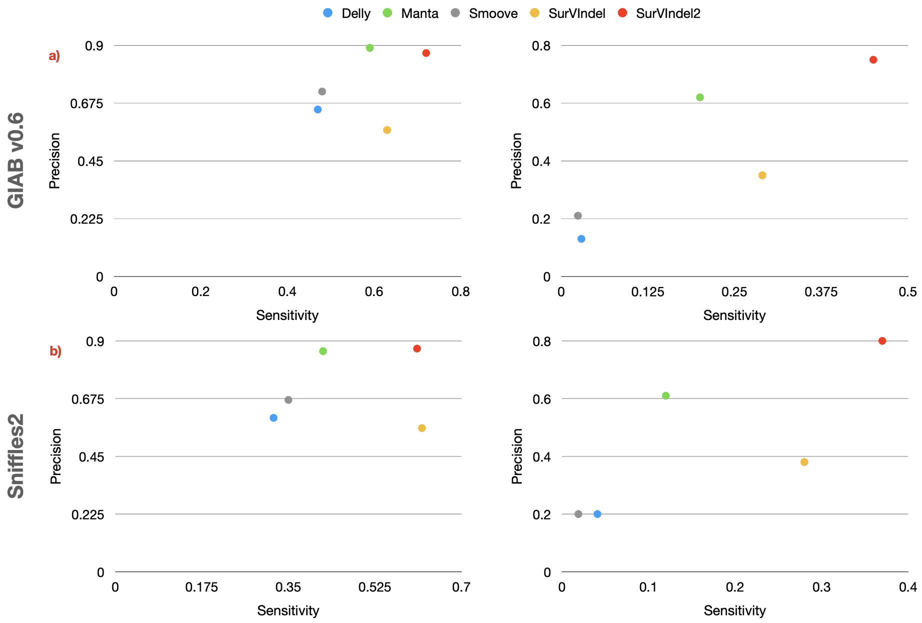

Supplementary Figure 7: **Additional benchmarking: Genome in a Bottle (GIAB) and Sniffles2 benchmarks for HG002.** (a) Sensitivity and precision for deletions (left) and tandem duplications (right) for the GIAB v0.6 benchmark. (b) Sensitivity and precision for deletions (left) and tandem duplications (right) for the Sniffles2 benchmark.

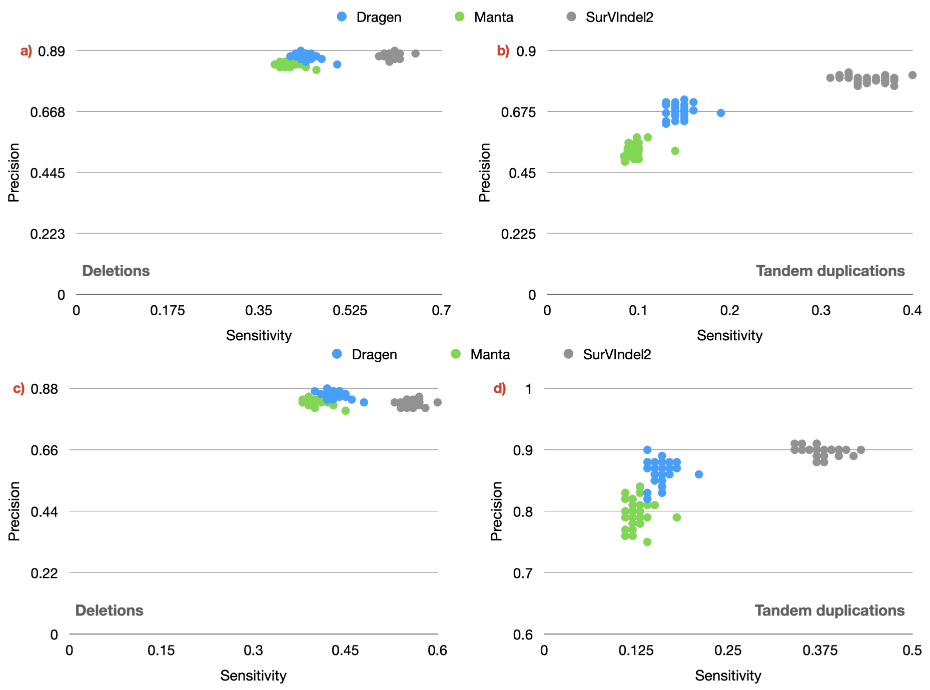

Supplementary Figure 8: **Additional benchmarking: performance of DRAGEN compared to Manta and SurVIndel2.** (a) Sensitivity and precision for deletions predicted by Manta, DRAGEN and SurVIndel2 on the HGSVC2 benchmark. (b) Similarly, sensitivity and precision for tandem duplications. Although DRAGEN exhibits clear improvements over Manta, SurVIndel2 remains superior in all metrics. (c, d) report the same metrics calculated by Truvari. The results largely match those reported by our comparison algorithm. It must be noted that both sensitivity and precision of SurVIndel2 are slightly lower, likely because it reports a higher number of deletions in repetitive regions, and Truvari does not employ a repeat-aware comparison algorithm (Methods). Furthermore, due to the aforementioned limitations in comparing insertions to tandem duplications, we have disabled the inserted sequence comparison, which causes methods to have higher precision for tandem duplications.

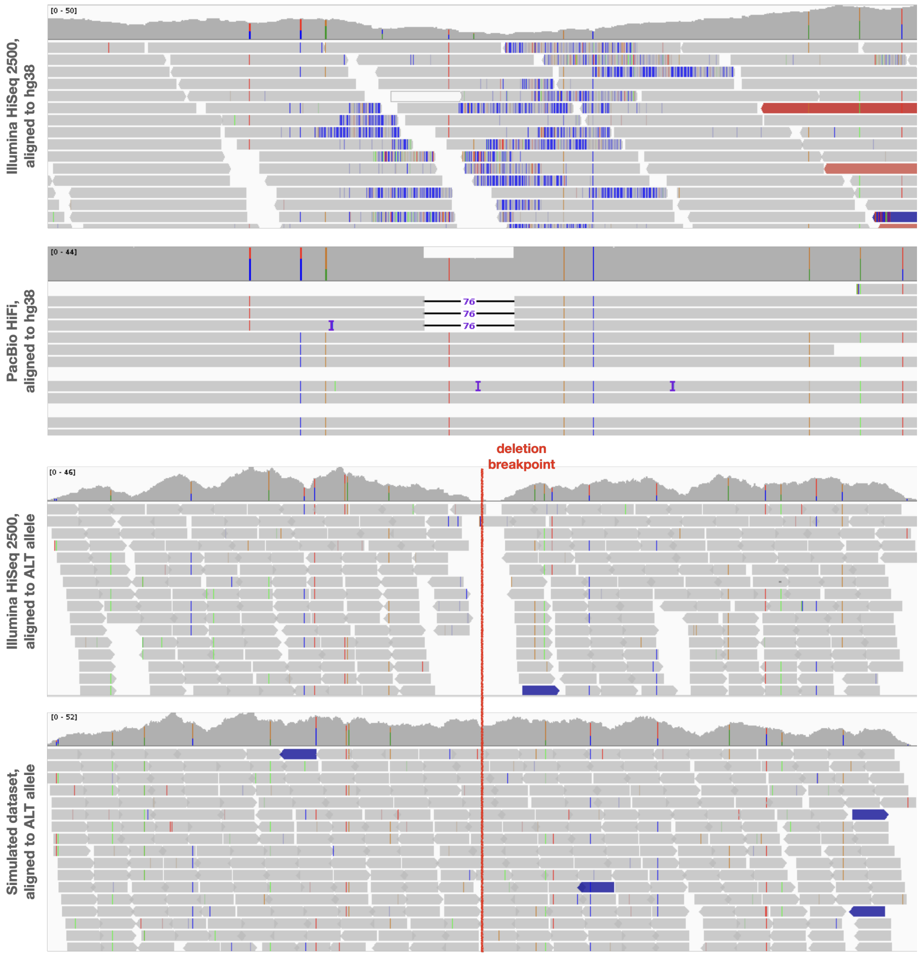

Supplementary Figure 9: **An example of unsupported deletion in the HGSVC2 catalogue for HG002, called chr10-729000-DEL-76.** The deletion is clearly supported by the HiFi reads aligned to hg38, but the HiSeq 2500 reads in the region appear to be very noisy. When aligned to the alternative allele, we observe that only one good quality read (BWA-MEM AS≥ 140, read length 150) contains the breakpoint. For this reason, despite having a high ES score (0.72), we cannot find reads supporting the deletion: for this reason, SurVIndel2 does not call it. However, in the simulated reads, plenty of correctly aligned reads contain the breakpoint. Unsurprisingly, SurVIndel2 can detect the deletion in the simulated dataset.

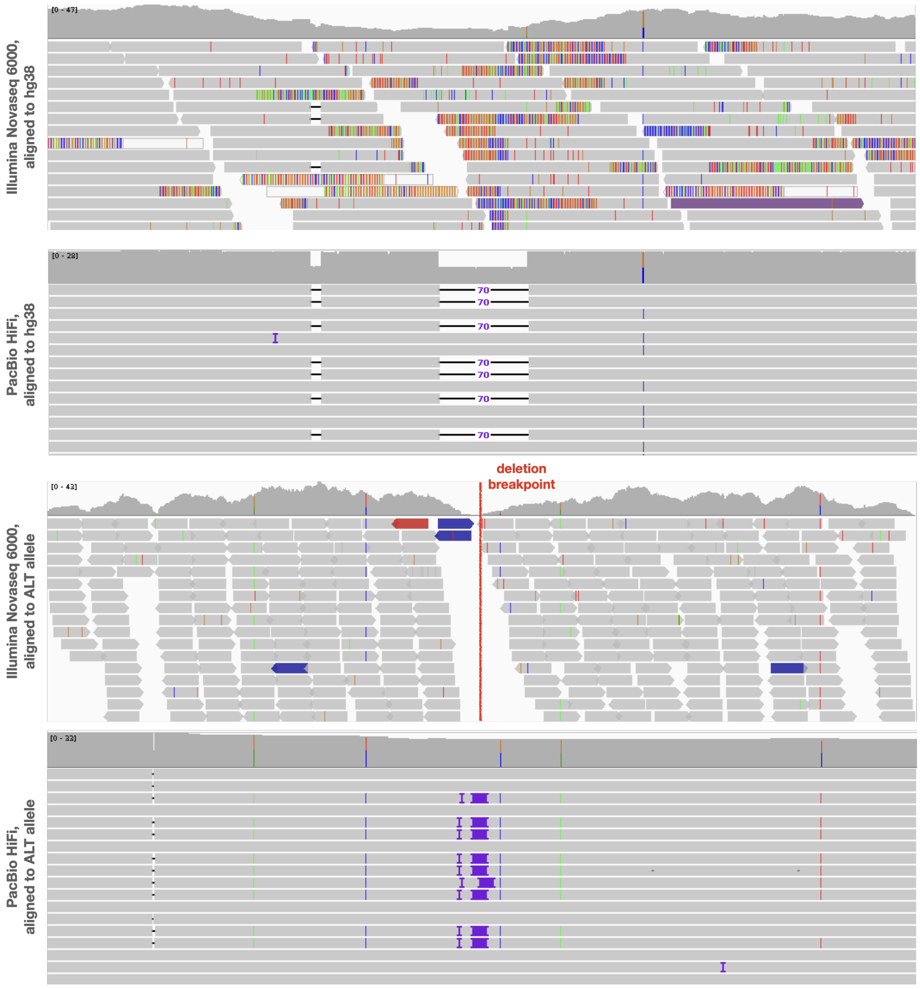

Supplementary Figure 10: **An example of unsupported deletion in the HGSVC2 catalogue for HG00512, called chr1-2262046-DEL-70.** The deletion is clearly supported by the HiFi reads aligned to hg38, but the Illumina reads in the region appear to be very noisy. When aligned to the alternative allele, we observe that there are no good quality reads (BWA-MEM AS$\geq$ 140, read length 150) that contain the breakpoint. For this reason, despite having a high ES score (0.633), we cannot find any reads supporting the deletion. Many HiFi reads perfectly align the the alternative allele, which confirms that the alternative allele is correct.

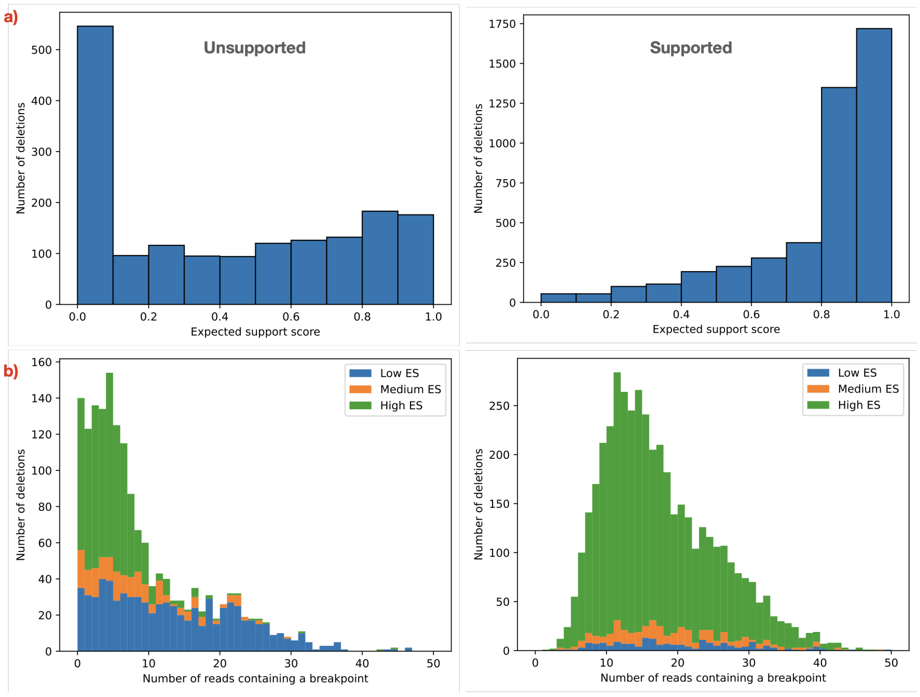

Supplementary Figure 11: **Factors that contribute to the absence of support for deletions in HG00512.** The results confirm our findings on HG002. a) As for HG002, a large portion of unsupported deletions have low expected support (ES) score. However, 44% of the unsupported deletions have high ES score (≥ 0.5), which suggest that there is another reason the lack of support. b) Another factor is an absence of correctly sequenced reads containing the breakpoint of the deletions.

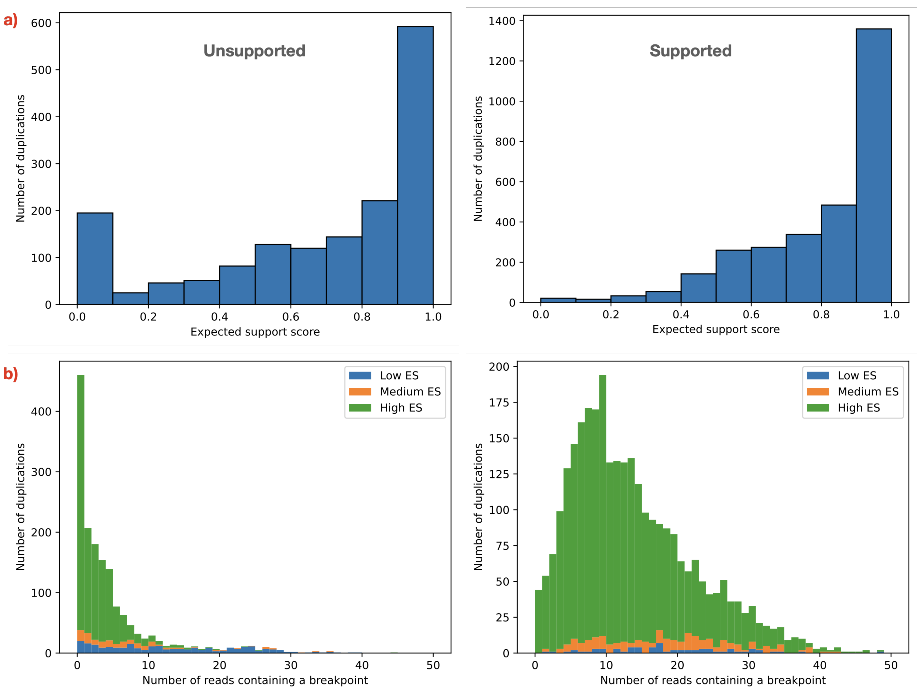

Supplementary Figure 12: **Factors that contribute to the absence of support for duplications in HG00512.** The results confirm our findings on HG002. a) As for HG002, most unsupported deletions (75%) have high expected support (ES) score (≥ 0.5), which suggest that there is another reason the lack of support. b) The absence of correctly sequenced reads containing the breakpoints of the duplication is even more prevalent than in deletions.

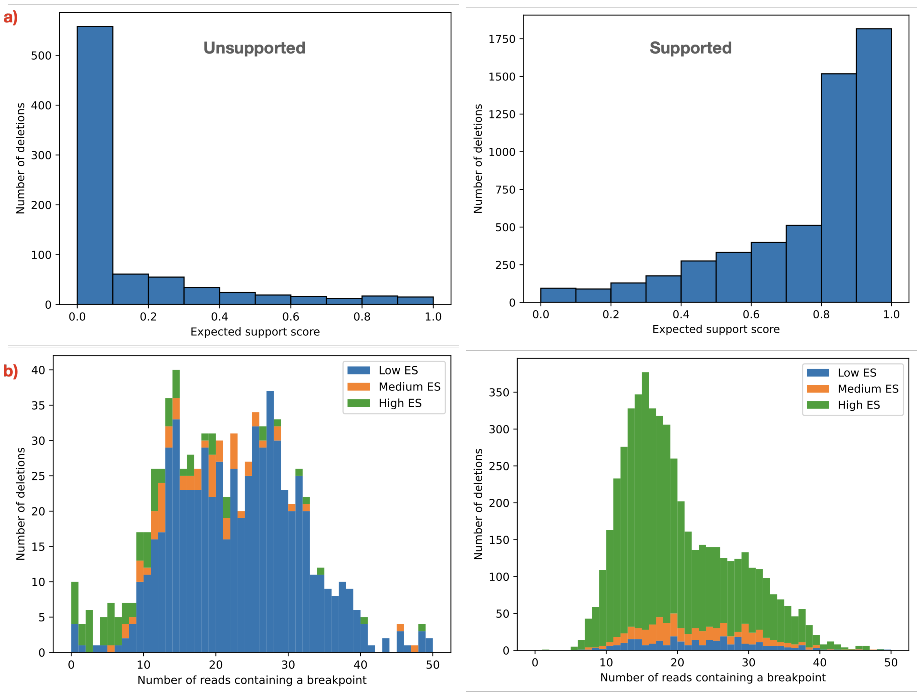

Supplementary Figure 13: **Factors that contribute to the absence of support for deletions, in a realistic synthetic dataset for HG002.** a) As expected, the vast majority of unsupported deletions have low expected support (ES) score, while supported deletions have high ES. Unlike the real dataset (Fig. 9), nearly no unsupported deletions have high ES. b) The real dataset exhibited a large number of deletions that have high ES but no correctly sequenced reads containing their breakpoints. This phenomenon is absent from the synthetic dataset.

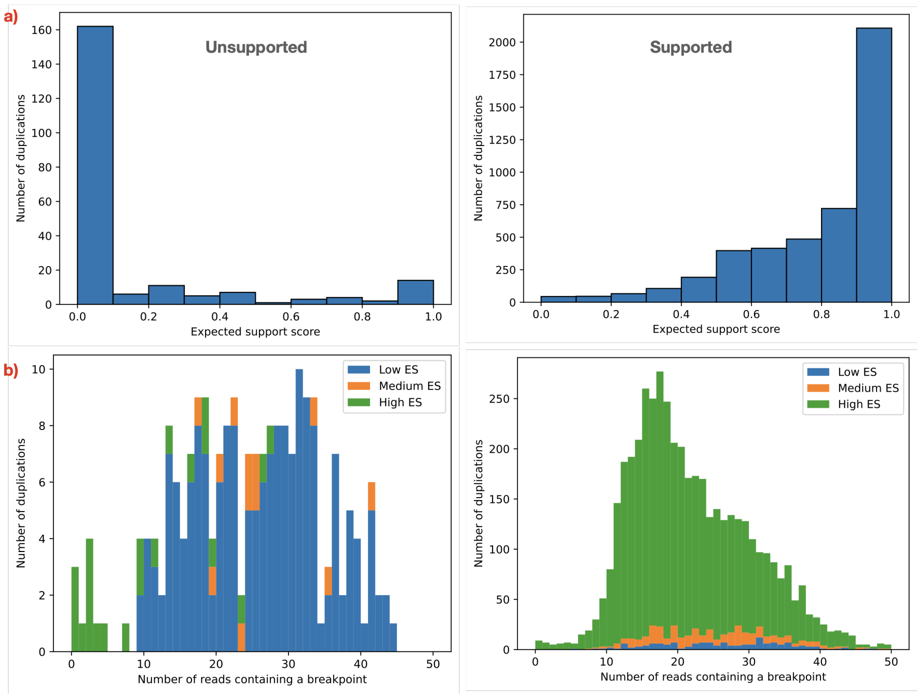

Supplementary Figure 14: **Factors that contribute to the absence of support for duplications, in a realistic synthetic dataset for HG002.** a) As expected, the vast majority of unsupported duplications have low expected support (ES), while supported duplications have high ES. This is in stark contrast to the real dataset, where the majority of unsupported duplications had high ES (Fig. 10). b) The real dataset exhibited a large number of duplications that have high ES but no correctly sequenced reads containing their breakpoints. This phenomenon is absent from the synthetic dataset.

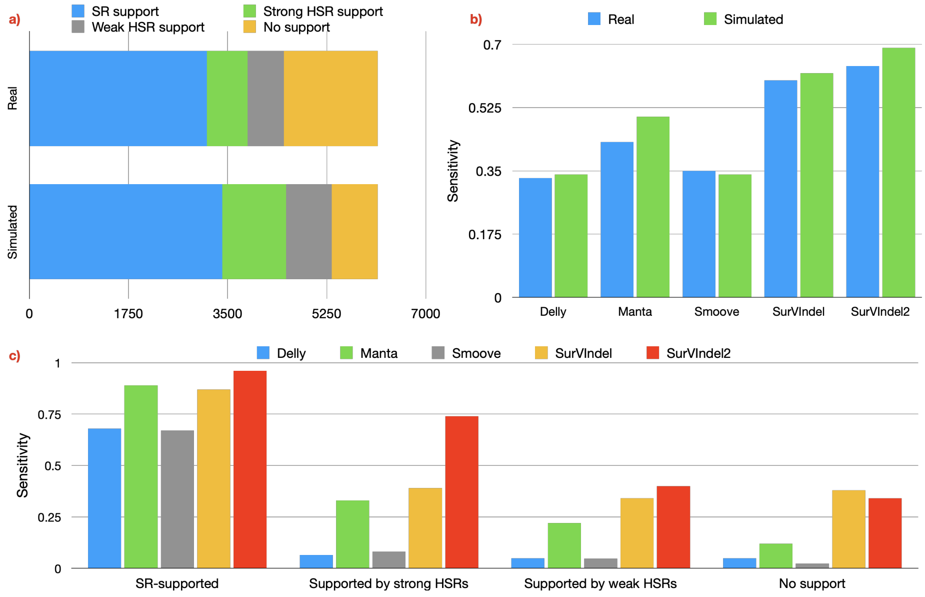

Supplementary Figure 15: **Support for deletions in the synthetic versus the real dataset.** a) The simulated dataset has less unsupported deletions. b) The sensitivity of some methods is increased. This is particularly true for Manta (increase from 0.43 to 0.5) and SurVIndel2 (increase from 0.64 to 0.69). c) However, when stratified by evidence available, the sensitivity of the methods for each category are very similar between the real (Fig. 4c) and the synthetic datasets. This, combined with Supplementary Fig. 13 shows that the increase in sensitivity is due to less unsupported deletions.

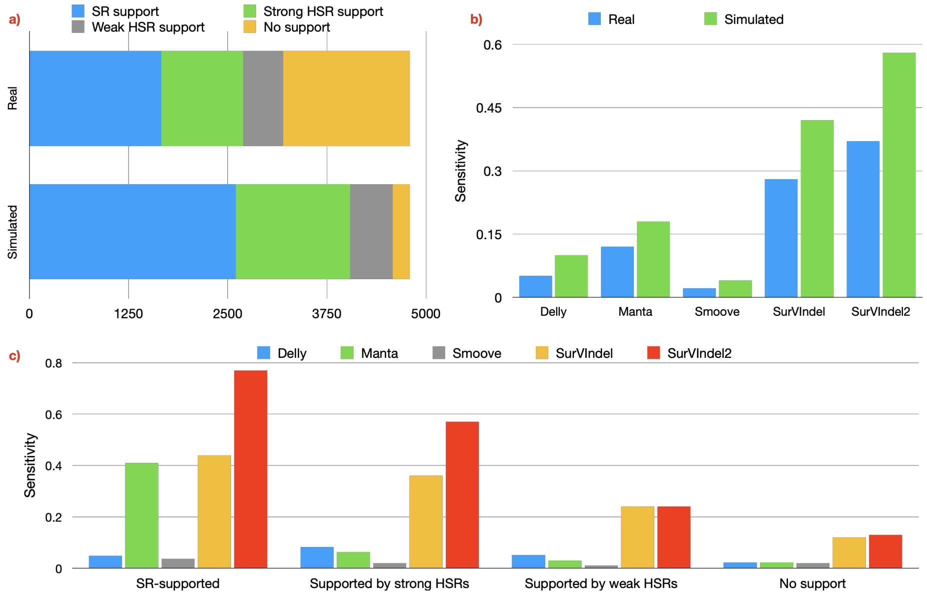

Supplementary Figure 16: **Support for tandem duplications in the synthetic versus the real dataset.** a) The simulated dataset has far less unsupported duplications. b) The sensitivity of all the methods is considerably increased. Particularly notable is the performance of SurVIndel2 improves from 0.37 to 0.58. c) Once again, when stratified by evidence available, the sensitivity of the methods for each category are very similar between the real (Fig. 4d) and the synthetic datasets. This, combined with Supplementary Fig. 14 shows that the methods are severely limited because the regions containing the tandem duplications are not sequenced correctly.

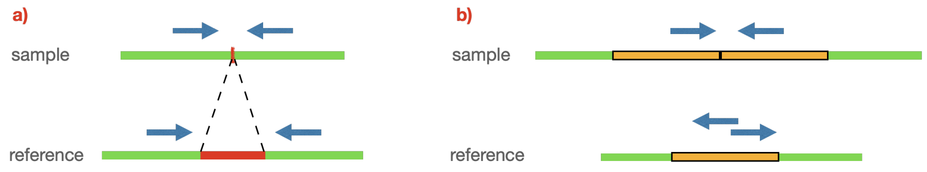

Supplementary Figure 17: (a) Consider a read pair s.t. one read was sequenced upstream and one downstream of the deletion breakpoint, sequenced from a fragment of size L. After mapping the pair to the reference, its insert size will be $L + d$, where d is the size of the deletion. If the deletion is large enough, the insert size will also be abnormally large, when compared to the insert size distribution of the library. (b) When a duplication is large, reads sequenced from different copies of the duplicated sequence may show the wrong relative orientation when aligned to the reference.

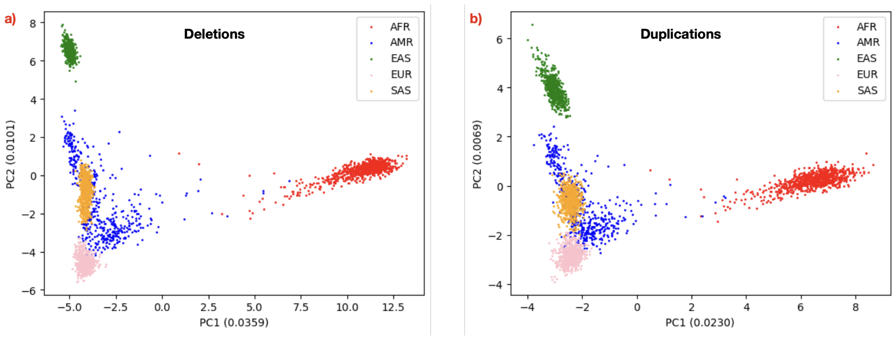

Supplementary Figure 18: **Principal Component Analysis (PCA) separated by copy number variant type.** PCA based on deletions (a) and tandem duplications (b). Both event types are able to clearly separate the superpopulations.

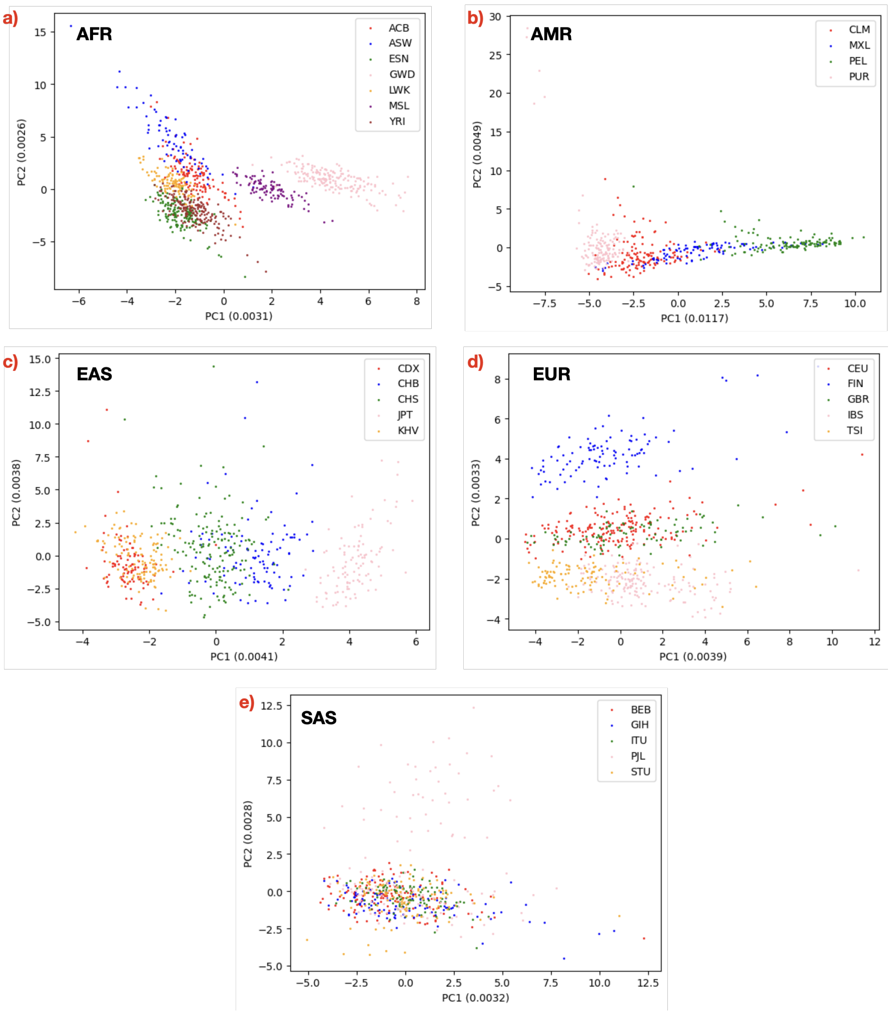

Supplementary Figure 19: **Principal Component Analysis (PCA) by subpopulation.** PCA based on both deletions and duplications was able to segregate each superpopulation into subpopulations. (a) For Africans (AFR), two subpopulations are clearly separated from the others: Mende in Sierra Leone (MSL) and Gambian in Western Division (GWD). (b) Americans (AMR) subpopulations are segregated on PC1, while PC2 is dominated by heterogeneity among Puerto Ricans (PUR). (c) East Asians (EAS) form three major clusters: (1) Vietnamese and Dai Chinese, (2) Han Chinese from Beijing with Southern Han Chinese and (3) Japanese. Some degree of separation between Han Chinese from Beijing and Southern Han Chinese is also observed. Similarly (d), Europeans (EUR) are also clustered into three clusters: (1) Finnish, (2) British and Utah residents with Northern and Western European ancestry and (3) Italians from Tuscany and Spanish. (e) South Asians (SAS) structure is much less obvious than other superpopulations. Furthermore, there appears to be high heterogeneity among Punjabi from Lahore (PJL). These results agree what we previously observed from insertions [2]

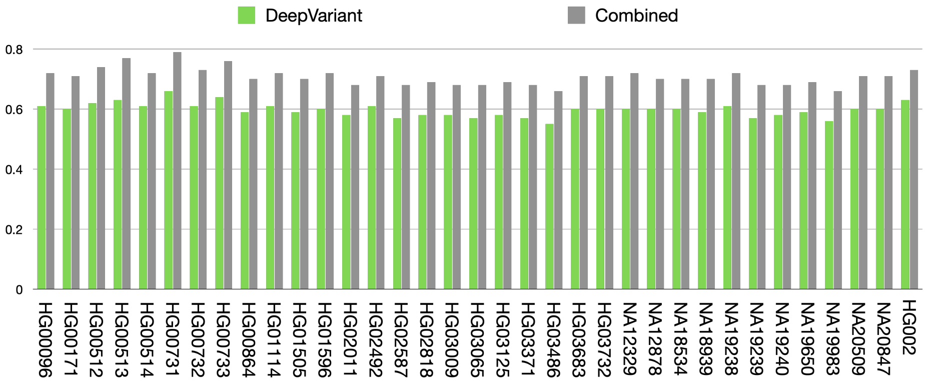

Supplementary Figure 20: **Sensitivity of DeepVariant and DeepVariant plus SurVIndel2 on the 35 HGSVC2 samples, for deletions between 30 and 50 bp.**

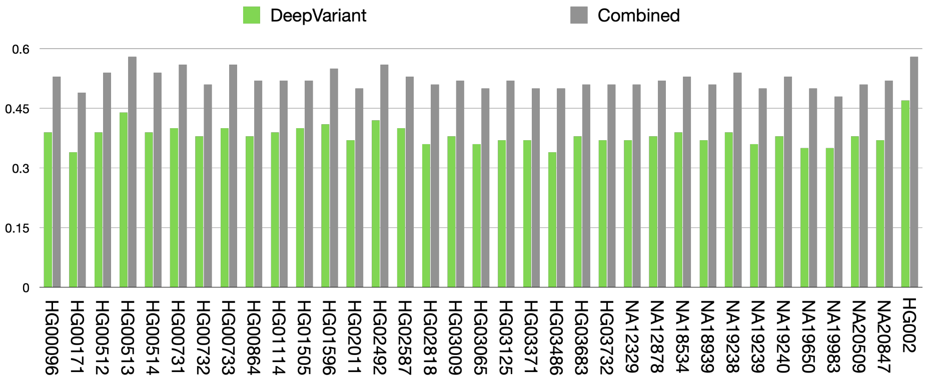

Supplementary Figure 21: **Sensitivity of DeepVariant and DeepVariant plus SurVIndel2 on the 35 HGSVC2 samples, for insertions between 30 and 50 bp.**

Supplementary Tables

	**HG002 DEL**	**HG002 DUP**	**HG00512 DEL**	**HG00512 DUP**
No reads	126	106	224	179
No SV	467	232	417	214
Multiple SVs	111	217	119	202
Incompatible SV	288	576	262	551
No good LR support	845	734	729	833
Other	45	58	41	44
High quality	6150	4796	6148	4585

Supplementary Table 1: **Number of high-quality alleles for deletions and duplications in HG002 and HG00512, as well as the number of alleles removed by each filter.** Each putative alternative allele undergoes a strict filtering process, in order to remove potentially incorrect sequences.

Supplementary References

[1] Rajaby, R., Sung., W.K. SurVIndel: improving CNV calling from high-throughput sequencing data through statistical testing. *Bioinformatics*, Volume 37, Issue 11, June 2021

[2] Rajaby, R., Liu D.X., Au C.H., Cheung Y.T., Lau A.Y.T., Yang. Q.Y., Sung., W.K. INSurVeyor: improving insertion calling from short read sequencing data *Nat Commun*, 14(1):3243, Jun 2023.
