# Supplementary material for: SurVIndel2: improving copy number variant calling from next-generation sequencing using hidden split reads
Source: Nat Commun. 2024 Dec 2;15:10473. doi: 10.1038/s41467-024-53087-7 (PMC11612505; doi:10.1038/s41467-024-53087-7)
Supplement: Supplementary file 3 — Reporting Summary [file 41467_2024_53087_MOESM3_ESM.pdf]

nature portfolio

Corresponding author(s): Wing-Kin Sung

Last updated by author(s): Ramesh Rajaby

Reporting Summary

Nature Portfolio wishes to improve the reproducibility of the work that we publish. This form provides structure for consistency and transparency in reporting. For further information on Nature Portfolio policies, see our Editorial Policies and the Editorial Policy Checklist.

Statistics

For all statistical analyses, confirm that the following items are present in the figure legend, table legend, main text, or Methods section.

n/a	Confirmed	
☐	☒	The exact sample size (*n*) for each experimental group/condition, given as a discrete number and unit of measurement
☒	☐	A statement on whether measurements were taken from distinct samples or whether the same sample was measured repeatedly
☒	☐	The statistical test(s) used AND whether they are one- or two-sided<br>*Only common tests should be described solely by name; describe more complex techniques in the Methods section.*
☒	☐	A description of all covariates tested
☒	☐	A description of any assumptions or corrections, such as tests of normality and adjustment for multiple comparisons
☐	☒	A full description of the statistical parameters including central tendency (e.g. means) or other basic estimates (e.g. regression coefficient) AND variation (e.g. standard deviation) or associated estimates of uncertainty (e.g. confidence intervals)
☒	☐	For null hypothesis testing, the test statistic (e.g. *F*, *t*, *r*) with confidence intervals, effect sizes, degrees of freedom and *P* value noted<br>*Give P values as exact values whenever suitable.*
☒	☐	For Bayesian analysis, information on the choice of priors and Markov chain Monte Carlo settings
☒	☐	For hierarchical and complex designs, identification of the appropriate level for tests and full reporting of outcomes
☒	☐	Estimates of effect sizes (e.g. Cohen's *d*, Pearson's *r*), indicating how they were calculated

Our web collection on statistics for biologists contains articles on many of the points above.

Software and code

Policy information about availability of computer code

Data collection	No software was used for data collection.
Data analysis	The data was mainly analysed with SurVIndel2: https://github.com/kensung-lab/SurVIndel2. RepeatMasker 4.1.2-p1 was used for repeat annotation. Delly 1.1.3, Manta 1.6.0, Smoove 0.2.8, and SurVIndel latest were run to provide a baseline for comparison. BWA MEM 0.7.17 and samtools were used to generate the BAM file for HG002, and for the different non-human organisms. SurVClusterer (https://github.com/Mesh89/SurVClusterer) was used to create the joint dataset of CNVs for 1000g and benchmarking the CNVs of the different callers. The version 1.5.0 of Deepvariant was used to test the complementarity with SurVIndel2. spoa 4.0.9 was used to build contigs from the the PacBio HiFi reads. ART MountRainier was used to simulate paired-end reads.

For manuscripts utilizing custom algorithms or software that are central to the research but not yet described in published literature, software must be made available to editors and reviewers. We strongly encourage code deposition in a community repository (e.g. GitHub). See the Nature Portfolio guidelines for submitting code & software for further information.

Data

Policy information about availability of data

All manuscripts must include a data availability statement. This statement should provide the following information, where applicable:

- Accession codes, unique identifiers, or web links for publicly available datasets
- A description of any restrictions on data availability
- For clinical datasets or third party data, please ensure that the statement adheres to our policy

The CNVs catalogue produced by SurVIndel2 for the 1000g project has been deposited at EMBL-EBI European Variation Archive, project ID PRJEB71638 (https://www.ebi.ac.uk/ena/browser/view/PRJEB71638). The IGV screenshots for manual validation of 60 reported false positive small CNVs (30 in HG002 and 30 in HG00512) have been deposited at Zenodo: https://zenodo.org/records/10811268.

Data, scripts, and instructions to reproduce the main figures in the paper have been deposited on GitHub: https://github.com/kensung-lab/survindel2_paper_experiments. Sequencing data for HG002 was downloaded from NCBI (accessions SRR1766442 to SRR1766486). PacBio HiFi data for HG002 was downloaded from NCBI under accession code PRJNA586863 (https://www.ncbi.nlm.nih.gov/bioproject/PRJNA586863).

The HG002 benchmark catalogue and the list of tier 1 regions were downloaded from https://ftp-trace.ncbi.nlm.nih.gov/ReferenceSamples/giab/data/AshkenazimTrio/analysis/NIST_SVs_Integration_v0.6/. Information on accessing the 3,202 CRAM files for the 1KGP project produced by NYGC can be found at https://www.internationalgenome.org/data-portal/data-collection/30x-grch38. The Phase 2 benchmark calls produced by HGSVC are available at https://www.internationalgenome.org/data-portal/data-collection/hgsvc2. The SV catalogue produced by NYGC was downloaded from http://ftp.1000genomes.ebi.ac.uk/vol1/ftp/data_collections/1000G_2504_high_coverage/working/20210124.SV_Illumina_Integration/1KGP_3202.gatksv_svtools_novelins.freeze_V3.wAF.vcf.gz.

Data from the 1001 Genomes Project was downloaded from NCBI under accession code PRJNA273563 (https://www.ncbi.nlm.nih.gov/bioproject/PRJNA273563). Accession codes for individual samples are: Alo-0 (ERR10084935 and SRR1946106), Cas-0 (ERR10084942 and SRR1946392), Cat-0 (ERR10084948 and SRR1946393), Cvi-0 (ERR10084078 and SRR1945758), Evs-0 (ERR10084952 and SRR1946405), Hom-4 (ERR10084958 and SRR1946144), and Hum-2 (ERR10084063 and SRR1946147).

All the data for the other organisms were downloaded from NCBI, and accessions will be provided in the following paragraphs. PacBio HiFi reads for the Bos taurus samples were downloaded from accessions ERR10378054 to ERR10378058, while two short read libraries, which were treated as independent samples, were downloaded from accessions ERR10310239 and ERR10310240.

PacBio HiFi reads for the Mus musculus sample were downloaded from accession SRR23686163, while short reads were downloaded from accession SRR23690179. PacBio HiFi reads for the Oryza sativa samples were downloaded from accession SRR10238608 (for MH63) and SRR13280199 (for ZS97), while short reads were downloaded from accession SRR13124689 (for MH63) and SRR13124696 (for ZS97).

Research involving human participants, their data, or biological material

Policy information about studies with human participants or human data. See also policy information about sex, gender (identity/presentation), and sexual orientation and race, ethnicity and racism.

Reporting on sex and gender	Neither sex nor gender were considered in the study.
Reporting on race, ethnicity, or other socially relevant groupings	We only considered the distribution of the number of SVs in each ethnic group. This was only to confirm that it followed the expected trends.
Population characteristics	The only population characteristics considered was ethnicity, both in the form of superpopulation (East Asian, European, African, Central/South American, South Asian) and subpopulation.
Recruitment	We used publicly available data.
Ethics oversight	We used publicly available data.

Note that full information on the approval of the study protocol must also be provided in the manuscript.

Field-specific reporting

Please select the one below that is the best fit for your research. If you are not sure, read the appropriate sections before making your selection.

☒ Life sciences ☐ Behavioural & social sciences ☐ Ecological, evolutionary & environmental sciences

For a reference copy of the document with all sections, see nature.com/documents/nr-reporting-summary-flat.pdf

Life sciences study design

All studies must disclose on these points even when the disclosure is negative.

Sample size	We aimed at demonstrating the performance of our caller on the largest public human dataset, the full 1000 Genome Project (3,202 samples).
Data exclusions	No data was excluded.
Replication	We ensured that running the software on the same sample multiple times would produced consistent results.
Randomization	Randomization was not applicable to any of the analysis or experiments performed.
Blinding	Blinding was not applicable to any of the analysis or experiments performed.

Reporting for specific materials, systems and methods

We require information from authors about some types of materials, experimental systems and methods used in many studies. Here, indicate whether each material, system or method listed is relevant to your study. If you are not sure if a list item applies to your research, read the appropriate section before selecting a response.

Materials & experimental systems

n/a	Involved in the study
☒	☐ Antibodies
☒	☐ Eukaryotic cell lines
☒	☐ Palaeontology and archaeology
☒	☐ Animals and other organisms
☒	☐ Clinical data
☒	☐ Dual use research of concern
☒	☐ Plants

Methods

n/a	Involved in the study
☒	☐ ChIP-seq
☒	☐ Flow cytometry
☒	☐ MRI-based neuroimaging

Plants

Seed stocks	*Report on the source of all seed stocks or other plant material used. If applicable, state the seed stock centre and catalogue number. If plant specimens were collected from the field, describe the collection location, date and sampling procedures.*
Novel plant genotypes	*Describe the methods by which all novel plant genotypes were produced. This includes those generated by transgenic approaches, gene editing, chemical/radiation-based mutagenesis and hybridization. For transgenic lines, describe the transformation method, the number of independent lines analyzed and the generation upon which experiments were performed. For gene-edited lines, describe the editor used, the endogenous sequence targeted for editing, the targeting guide RNA sequence (if applicable) and how the editor was applied.*
Authentication	*Describe any authentication procedures for each seed stock used or novel genotype generated. Describe any experiments used to assess the effect of a mutation and, where applicable, how potential secondary effects (e.g. second site T-DNA insertions, mosiacism, off-target gene editing) were examined.*
